# Regulatory Mechanisms of lncRNAs and Their Target Gene Signaling Pathways in Laryngeal Squamous Cell Carcinoma

**DOI:** 10.3389/fphar.2020.01140

**Published:** 2020-07-27

**Authors:** Wei Li, Yu Chen, Xuqiang Nie

**Affiliations:** ^1^ College of Pharmacy, Zunyi Medical University, Zunyi, China; ^2^ Joint International Research Laboratory of Ethnomedicine of Chinese Ministry of Education, College of Pharmacy, Zunyi Medical University, Zunyi, China

**Keywords:** lncRNA, laryngeal squamous cell carcinoma, signaling pathways, Wnt/*β*-catenin pathway, cancer

## Abstract

Laryngeal squamous cell carcinoma (LSCC) is a common malignant tumor that occurs in the head and neck. People living in areas with serious air pollution and those who smoke and drink for a long time belong to high-risk groups. Although great progress has been made in chemotherapy, radiotherapy, and molecular targeted therapy in recent years, the prognosis of patients is still not good. The proliferation, invasion, and apoptosis of LSCC are controlled by many factors, which are the key factors influencing the prognosis of patients. Previous researches have demonstrated that long noncoding RNAs (lncRNAs) can be used as oncogenes or tumor suppressor genes in the occurrence and development of cancer and regulate cancer through various ways including epigenetic regulation and post-transcriptional regulation. The characteristics and roles of lncRNAs in LSCC, however, are not clear. In this review, we will discuss the role and function of lncRNAs in the proliferation, invasion, and apoptosis of LSCC and analyze the relationship between lncRNAs and lncRNA-regulated signaling pathways in LSCC pathological process. The difficulties faced by the related research of LSCC are discussed. It provides reference ideas for the molecular mechanism research of LSCC targeting lncRNA and its signaling pathways, the development of clinical prevention and therapeutic drug and individualized treatment, thereby improving the quality of life of patients.

## Introduction

Laryngeal squamous cell carcinoma (LSCC) is one of the most frequent malignant neoplasms in the head and neck. At present, the treatment strategies of LSCC include laryngectomy, radiotherapy and chemotherapy, or a combination of multiple therapies. Despite the development of improved strategies and more accurate treatments, the 5-year survival rate of laryngeal squamous cell carcinoma has unfortunately dropped from 66 to 63% in the past 40 years (Steuer et al., 2017). The larynx plays a key role in breathing, swallowing, and vocalization, and surgical resection has a great influence on the quality of life for patients in the later stage. Therefore, early detection of LSCC, exploration of molecular markers of LSCC, and new therapeutic targets are crucial.

In recent years, with the development of large-scale high-throughput chips and second-generation sequencing technology, the relationship between lncRNAs and tumor-related genes has attracted more and more attention. lncRNAs are a kind of ribonucleic acid molecules with transcripts of more than 200 bp that do not encode proteins. Like messenger RNA, they can be transcribed by RNA polymerase II (Bhan et al., 2017). lncRNAs can regulate gene expression at various levels including pretranscription, posttranscription, and protein translation and are widely distributed in mammals (Guttman et al., 2009). Previous research indicated that lncRNAs are closely related to the occurrence and development of tumors. Changes in lncRNAs can participate in tumor chromatin remodeling and transcriptional regulation and play an important role in tumorigenesis and development (Pandey et al., 2008; Gupta et al., 2010; Kogo et al., 2011). Accumulating evidence suggests the importance of lncRNAs in human cancer, but their role and mechanism in LSCC are still unclear. Therefore, the research on the relationship between lncRNAs and LSCC can provide a new guiding strategy for the early diagnosis and individualized treatment of LSCC, which is of great significance. This article will briefly review the research progress of lncRNA in LSCC.

## LncRNA and LSCC Proliferation, Migration, Invasion, and Apoptosis

Proto-oncogenes are genes related to cell proliferation, which are necessary to maintain the normal life activities of the body, and are highly conserved in evolution. Tumor suppressor genes, also known as anticancer genes, exist in normal cells, and when activated, they can inhibit cell proliferation. Under normal circumstances, they play an important role in regulating the development, growth, and differentiation of cells. When proto-oncogenes or tumor suppressor genes are abnormally expressed or mutated, the quantity or activity of gene products changes, which may form tumors.

**Figure 1:**
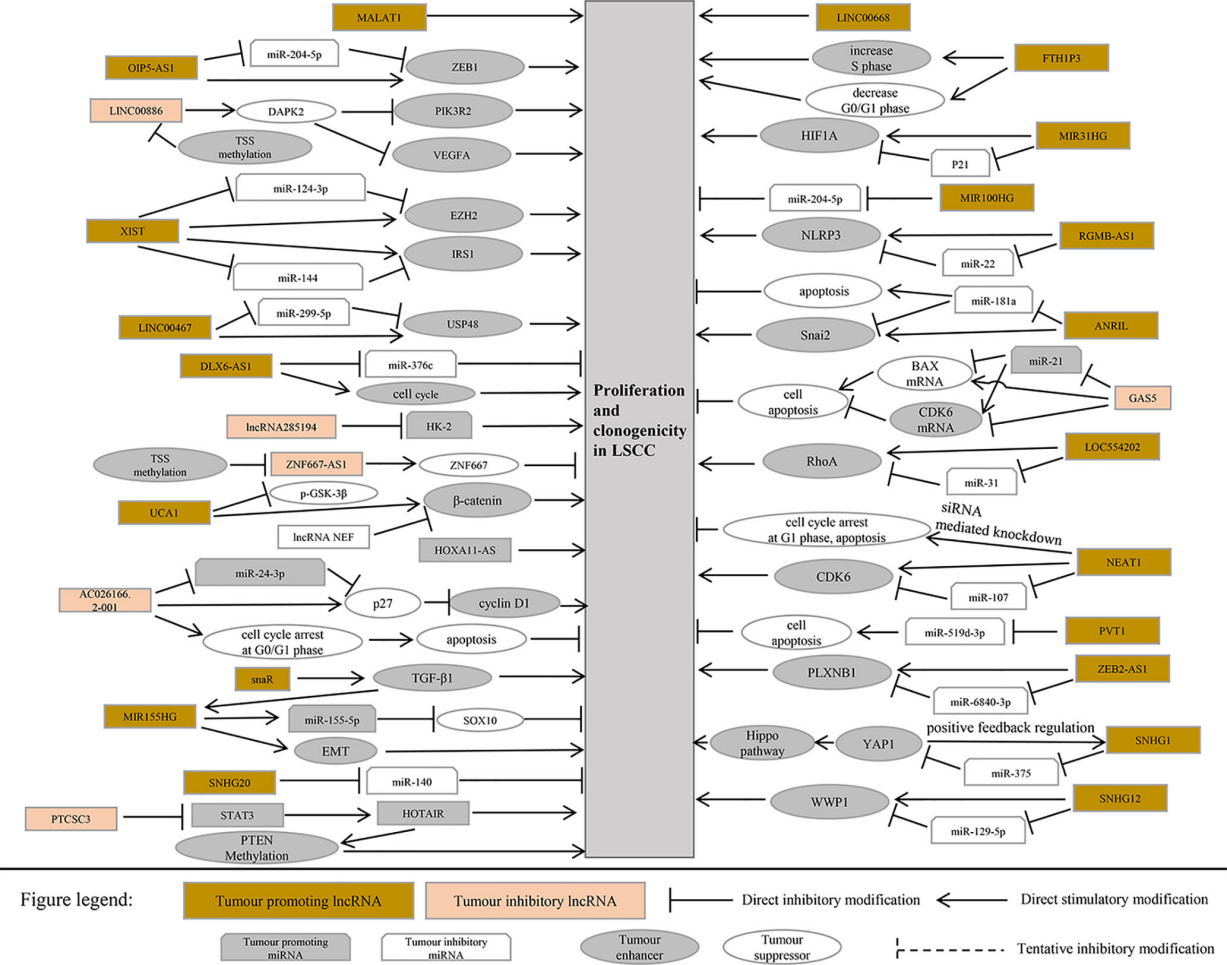
LncRNA related to proliferation and apoptosis in laryngeal squamous cell carcinoma.

**Figure 2:**
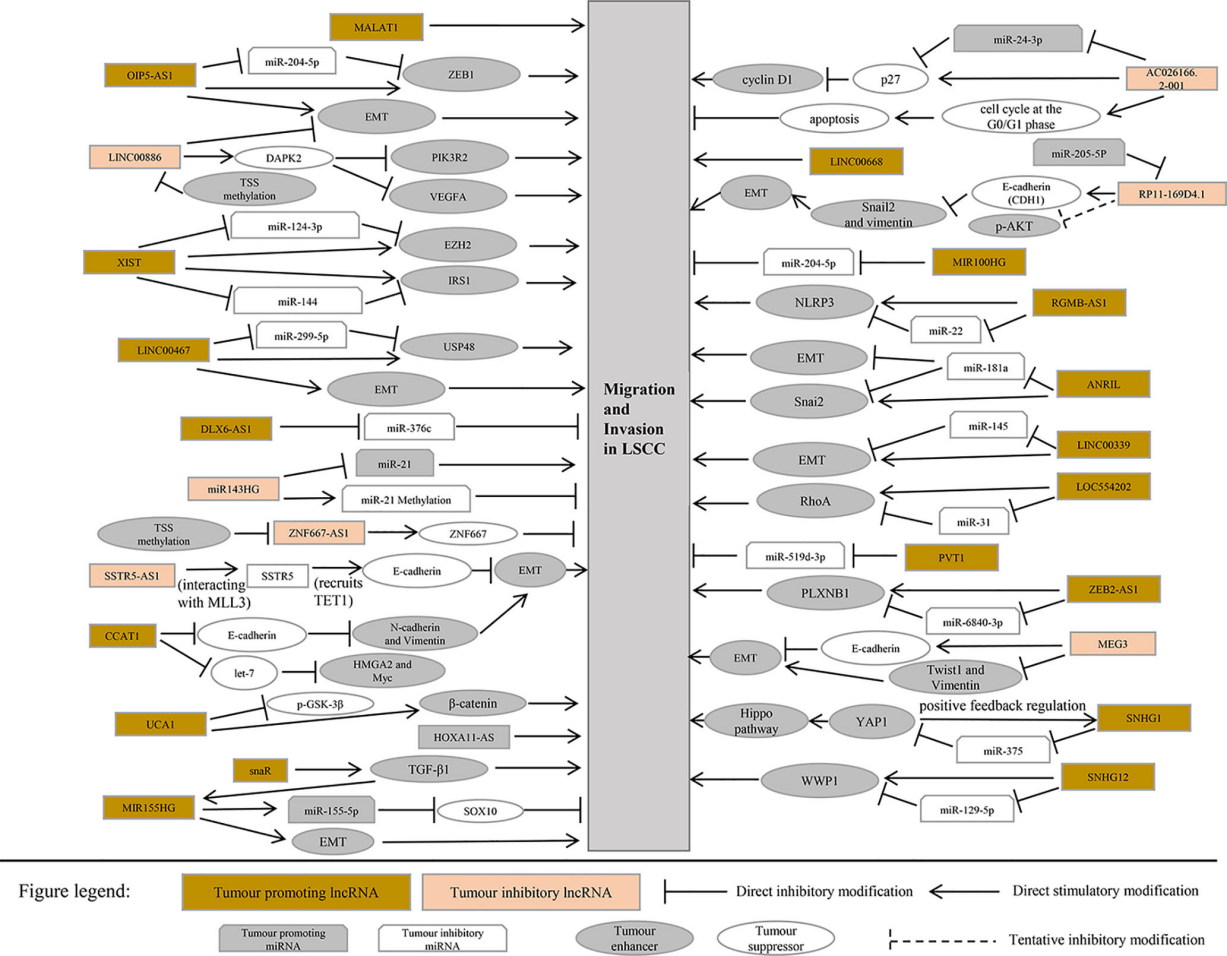
LncRNA associated with migration and invasion of laryngeal squamous cell carcinoma.

This section summarizes the relationship between dysregulated lncRNA in LSCC and proliferation, invasion, migration, and apoptosis. In addition, the regulatory mechanisms involved in lncRNA are described in detail in **Figures 1** and **2**, **Tables 1** and **2**).

**Table 1:** LncRNA related to proliferation and apoptosis in laryngeal squamous cell carcinoma

lncRNA	Cells	Tissues	Expression in LSCCs	Related gene	Functional role	References (PMID)
lncRNA NEAT1	HEp-2	LSCC	Upregulated	miR-107/CDK6	Promote the proliferation of LSCC cells	26822763
lncRNA SNHG20	AMC-HN-8, HEp-2	LSCC	Upregulated	miR-140	The ability of cell proliferation was significantly enhanced in overexpression group	31081112
lncRNA HOTAIR	HEp-2	LSCC	Upregulated	PTEN	The ability of cell proliferation was significantly enhanced in overexpression group	23141928
lncRNA PTCSC3	UM-SCC-17A	LSCC	Downregulated	lncRNA HOTAIR	Overexpression of PTCSC3 mediates inhibition of LSCC cell proliferation	31171714
lncRNA ZEB2-AS1	Tu 212, HEp-2, 16HBE	LSCC	Upregulated	miR-6840-3p/PLXNB1	Promote proliferation, migration and invasion, which is related to poor prognosis	31564916
lncRNA GAS5	SNU-899, SNU-1076	LSCC	Downregulated	miR-21	Gas5 inhibits the progress of LSCC by negatively regulating miR-21, and its targets are involved in cell proliferation and apoptosis	31572003
lncRNA ZNF667-AS1	AMC-HN-8, Tu 177, Tu 212, Tu 686	LSCC	Abnormal methylation, Downregulated	ZNF667	It is a tumor suppressor gene that reduces the ability of cell proliferation	30684967
lncRNA Dlx6-AS1	HEp-2	LSCC	Upregulated	miR-376c	Promote cell proliferation, cycle, and invasion	31814904
lncRNA OIP5-AS1	16HBE, AMC-HN-8, Tu 177	LSCC	Upregulated	miR-204-5p/ZEB1	Promote the proliferation of LSCC cells	32009213
lncRNA MALAT1	Tu 686, Tu 177, AMC-HN-8, LSC-1, NHBEC	LSCC	Upregulated		Inhibit apoptosis	31837057/31792655/25257554
LINC00886	Tu 686, AMC-HN-8, Tu 177, 293T	LSCC	Downregulated	VEGFA/PI3K/AKT signaling pathway/EMT	Overexpression of INC00886 significantly reduced cell proliferation, migration, and invasion *in vitro* and inhibited tumor growth *in vivo*	32111441
LINC00467	HN6, SCC25, HN4, Cal27, SCC4		Upregulated	miR-299-5p/USP48	Growth, migration, and EMT processes	32159247
lncRNA 285194	UM-SCC-17A, HuLa-PC	LSCC	Downregulated	HK-2 mRNA	Inhibition of HK-2 expression and proliferation of LSCC cells	31555348
lncRNA CCAT1	HEp-2	LSCC	Upregulated	E-cadherin/N-cadherin/Vimentin, LET-7/HMGA2/Myc	Promote HEp-2 cell proliferation, colony formation, and cell cycle	27830017
lncRNA UCA1	AMC-HN-8	LSCC	Upregulated		Activation of Wnt/*β*-catenin signal pathway promotes the proliferation of LSCC cells	30679991
lncRNA NEF	UM-SCC-17A	LSCC	Downregulated		Inhibition of Wnt/*β*-catenin signal pathway inhibits proliferation and promotes apoptosis of LSCC cells	31186702
lncRNA HOXA11-AS	AMC-HN-8, HEp-2	LSCC	Upregulated		Promote the growth of LSCC cells	29511452
lncRNA AC026166.2-001	AMC-HN-8, TU-212		Downregulated	miR-24-3p/p27	It can inhibit the proliferation and promote apoptosis of LSCC cells *in vitro* and *in vivo*	29463827/25243407
LINC00668	Tu 177, Tu 212, Tu 686, AMC-HN-8	LSCC	Upregulated		Promote the ability of proliferation	30415008
lncRNA FTH1P3	HEp-2, Tu 212	LSCC	Upregulated		It promoted the proliferation of LSCC cells and inhibited the apoptosis	31142627
lncRNA MIR31HG		LSCC	Upregulated	HIF1A–P21	Promote cell cycle progression and inhibit apoptosis and promote HNSCC cell proliferation and tumorigenesis through HIF1A and p21	30458787
lncRNA MIR100HG	UM-SCC-17A	LSCC	Upregulated	miR-204-5p	Promote the proliferation of LSCC cells	31114240
lncRNA RGMB-AS1	HEp-2, AMC-HN-8	LSCC	Upregulated	miR-22/NLRP3 Pathway	Promote the proliferation of LSCC cells *in vitro* and tumor growth *in vivo*	31351424
lncRNA XIST	HEp-2, HEK293T	LSCC	Upregulated	miR-124/EZH2, miR-144/IRS1	Promote the ability of proliferation and colony formation	31071316/31894287
lncRNA ANRIL	AMC-HN-8, SNU-899	LSCC	Upregulated	miR-181a/Snai2	Promote the proliferation, clone formation, and apoptosis of LSCC cells	31667207
lncRNA LOC554202	Hep2	LSCC	Upregulated	miR-31/RhoA	Promote the growth and cell cycle of LSCC cells	29737563
lncRNA PVT1	Tu 686, Tu 177, LSC-1	LSCC	Upregulated	miR-519d-3p	Promote LSCC cell proliferation and inhibit apoptosis	30304557
lncRNA snaR	UM-SCC-17A	LSCC	Upregulated	TGF-*β*1	Promote the proliferation of LSCC cells	30506952
lncRNA MIR155HG	Tu 686, Tu 177, AMC-HN-8, 293T	LSCC	Upregulated	miR-155-5p/Sox10	Promote the progression of LSCC	31081043
lncRNA SNHG12	AMC-HN-8	LSCC	Upregulated	miR-129-5p/WWP1	Increase the proliferation of LSCC cells	31348766
lncRNA SNHG1	AMC-HN-8, HEp-2	LSCC	Upregulated	miR-375/YAP1/HIPPO Pathway	Promote growth and transfer *in vivo*	30323965

**Table 2:** LncRNA associated with migration and invasion of laryngeal squamous cell carcinoma.

lncRNA	Cells	Tissues	Expression in LSCCs	Related gene	Functional role	References (PMID)
lncRNA MALAT1	Tu 686, Tu 177, AMC-HN-8, LSC-1, NHBEC	LSCC	Upregulated		Promote migration and invasion, promote tumor cell metastasis	31837057/31792655/25257554
LINC00886	Tu 686, AMC-HN-8, Tu 177, 293 T	LSCC	Downregulated	VEGFA/PI3K/AKT signaling pathway/EMT	Significantly reduced the migration and invasion of cells *in vitro*	32111441
LINC00467	HN6, SCC25, HN4, Cal27, SCC4		Upregulated	miR-299-5p/USP48	Migration and EMT proc	32159247
lncRNA miR143HG	UM-SCC-17A	LSCC	Downregulated	miR-21	Inhibition of migration and invasion rate	31872875
lncRNA Dlx6-AS1	HEp-2	LSCC	Upregulated	miR-376c	Promote cell proliferation, cycle and invasion	31814904
lncRNA SSTR5-AS1	AMC-HN-8, Tu 212, Tu 686, Tu 177	LSCC	Downregulated	MLL3/H3K4me3	Upregulate E-cadherin and inhibit EMT	31196171
lncRNA CCAT1	HEp-2	LSCC	Upregulated	E-cadherin/N-cadherin/Vimentin, LET-7/HMGA2/Myc	Promote HEp-2 cell migration and invasion	27830017
lncRNA UCA1	AMC-HN-8	LSCC	Upregulated		Promote the invasion and migration of LSCC	30679991
lncRNA HOXA11-AS	AMC-HN-8, HEp-2	LSCC	Upregulated		Downregulation could significantly inhibit the growth, migration, and invasion of LSCC cells	29511452
LINC00668	Tu 177, Tu 212, Tu 686, AMC-HN-8	LSCC	Upregulated		Promote migration and invasion	30415008
lncRNA RP11-169D4.1	SNU-899, SNU-46	LSCC	Downregulated	miR-205-5p/RP11-169D4.1/Cdh1/AKT signaling pathway	Reduced the ability of cell migration and invasion *in vitro*	28534968
lncRNA AC026166.2-001	AMC-HN-8, TU-212		Downregulated	miR-24-3p/p27	Inhibit cell migration	29463827/25243407
lncRNA FTH1P3	HEp-2, Tu 212	LSCC	Upregulated		Promote the migration and invasion of LSCC cells	31142627
lncRNA OIP5-AS1	16HBE, AMC-HN-8, Tu 177	LSCC	Upregulated	miR-204-5p/ZEB1	Promote migration and invasion, promote EMT process	32009213
lncRNA MIR100HG	UM-SCC-17A	LSCC	Upregulated	miR-204-5p	Promote the migration and invasion of LSCC cells	31114240
lncRNA RGMB-AS1	HEp-2, AMC-HN-8	LSCC	Upregulated	miR-22/NLRP3	Promote of LSCC cell invasion *in vitro*	31351424
lncRNA ANRIL	AMC-HN-8, SNU-899	LSCC	Upregulated	miR-181a/Snai2	Promote LSCC cell invasion, migration, and EMT, to promote apoptosis	31667207
lncRNA LINC00339	Hep2, Tu 212	LSCC	Upregulated	miR-145	Promote LSCC cell invasion and EMT process	30485513
lncRNA LOC554202	HEp-2	LSCC	Upregulated	miR-31/RhoA	Promote the invasion of LSCC cells	29737563
lncRNA XIST	HEp-2, HEK293T	LSCC	Upregulated	miR-124/EZH2, miR-144/IRS1	Promote migration and invasion	31071316/31894287
lncRNA PVT1	Tu 686, Tu 177, LSC-1	LSCC	Upregulated	miR-519d-3p	Promote LSCC cell migration	30304557
lncRNA MEG3	HEp-2	LSCC	Downregulated	Twist1/Vimentin	Promote cell migration, invasion and EMT process	30915750
lncRNA snaR	UM-SCC-17A	LSCC	Upregulated	TGF-β1	Promote the migration and invasion of LSCC cells	30506952
lncRNA MIR155HG	Tu 686, Tu 177, AMC-HN-8, 293T	LSCC	Upregulated	miR-155-5p/Sox10	Promote the progress of LSCC and EMT	31081043
lncRNA SNHG12	AMC-HN-8	LSCC	Upregulated	miR-129-5p/WWP1	Increase the invasive ability of LSCC cells	31348766
lncRNA SNHG1	AMC-HN-8, HEp-2	LSCC	Upregulated	miR-375/YAP1/HIPPO signaling pathway	Increase *in vivo* metastasis	30323965
lncRNA Dlx6-AS1	HEp-2	LSCC	Upregulated	miR-376c	Promote cell proliferation, cycle and invasion	31814904

### LncRNA and EMT Process

Epithelial–mesenchymal transition (EMT) refers to the biological process in which epithelial cells are transformed into mesenchymal phenotype cells by specific procedures. The occurrence of EMT is mainly marked by the loss of E-cadherin in the epithelium and the increased expression of N-cadherin in the stroma. This cadherin switch leads to a drastic change in the adhesive properties of a cell, as it loses its affinity for epithelial neighbors and gains affinity for mesenchymal cells. Besides the change of the adhesive repertoire, the gain of N-cadherin expression also provokes increased cell migration and invasion (Yilmaz and Christofori, 2009).

The OIP5-AS1, an intergenic lncRNA transcribed in antisense orientation of OIP5 gene is overexpressed in LSCC tissues and cell lines. In addition, Human LSCC cell lines [AMC-HN-8 (RRID: CVCL_5966), Tu 177 (RRID: CVCL_4913)] and human bronchial epithelial cell line 16HBE were selected for *in vitro* test. The results revealed that overexpression of OIP5-AS1 promoted the proliferation, migration, invasion, and EMT process of LSCC cells. OIP5-AS1 was confirmed to serve as a competing endogenous RNA (ceRNA) of miR-204-5p in LSCC cells. ZEB1 is a target gene of miR-204-5p in LSCC. The restoration of miR-204-5p expression counteracts the carcinogenic effect mediated by OIP5-AS1 (Wang et al., 2020).

It has uncovered that the transcription of LINC00886 was significantly suppressed in tumor tissues and LSCC cell lines. The major cause for expression under LINC00886 in Human LSCC cell lines (AMC-HN-8, Tu 177) is abnormal methylation of LINC00886 transcriptional start site (TSS). Overexpression of LINC00886 greatly reduces cell proliferation, migration, and invasion *in vitro* and led to the strengthening of the G1 phase in AMC-HN-8 and Tu 177 cells. LINC00886 may inhibit the EMT process by upregulating E-cadherin meanwhile downregulating N-cadherin, Vimentin, and *β*-catenin in LSCC (Lan et al., 2020).

Inhibition of ZNF667-AS1 transcription was detected in LSCC cell lines and tumor tissues. It was also discovered that the CpG site of ZNF667-AS1 close to TSS was aberrant hypermethylated, which led to gene silencing. Overexpression of ZNF667-AS1 decreased the proliferation, migration, and invasion of AMC-HN-8 and Tu 177 cells. The sense strand ZNF667 is positively correlated with ZNF667-AS1 in expression and function. The overexpression of ZNF667-AS1 leads to the increase of ZNF667 expression at mRNA and protein level. In addition, research indicates that the role of ZNF667-AS1 and ZNF667 as tumor suppressor genes is related to the EMT process (Meng et al., 2019).

LncRNA SSTR5 is downregulated in LSCC tissues and cells. Aberrant DNA hypermethylation of the CpG sites clustered in the exon 1 and accumulation of inactive histone modifications at SSTR5 promoter region may be epigenetic mechanisms for SSTR5 inactivation in LSCC. SSTR5-AS1 was positively correlated with the expression and function of SSTR5 in LSCC. It is modulated by the methylation of the same CpG site of SSTR5. Overexpression of SSTR5-AS1 inhibits the proliferation, migration, and invasion of Human LSCC cell lines [AMC-HN-8, Tu 177 Tu 212 (RRID: CVCL_4915) and Tu 686 (RRID: CVCL_4916)]. More importantly, SSTR5-AS1 increases the enrichment of MLL3 and H3K4me3 in the SSTR5 promoter region by interacting with MLL3 and further induces SSTR5 transcription. In addition, overexpression of SSTR5-AS1 significantly increased the enrichment of TET1 in the E-cadherin promoter region. Overexpression of SSTR5-AS1 increases the mRNA and protein expression of E-cadherin and relieves the mRNA and protein expression of Vimentin to inhibit the EMT process (Wang et al., 2019).

The expression level of lncRNA MEG3 in tumor tissues of patients with LSCC was considerably lower than that in noncancerous tissues. *In vitro*, knockout of MEG3 gene in HEp-2 (RRID: CVCL_1906) cells greatly upgraded cell proliferation, migration, invasion, and EMT process by upregulating Twist1 and Vimentin and reducing E-cadherin (Zhao Y. Q. et al., 2019). Twist is also upregulated during early embryogenesis, tissue fibrosis, and tumor metastasis. In metastatic malignant tumor cells formed by type 3 EMT, Twist independently inhibits E-cadherin expression and promotes the expression of fibronectin and N-cadherin.

LncRNA microarray analysis of LSCC tissue showed that the transcription of AC026166.2-001 and RP11-169D4.1-001was abnormal (Shen et al., 2014). AC026166.2-001 can be used as a tumor suppressor in the progression of LSCC both *in vitro* and *in vivo*. The upregulation of AC026166.2-001 in AMC-HN-8 and Tu 212 cells induced cell cycle arrest in the G0/G1 phase, inhibited the proliferation and migration of LSCC cells, and upgraded apoptosis. In addition, AC026166.2-001 can inhibit the growth and induce apoptosis of LSCC xenografts in mice (Shen et al., 2018). Zhao et al. evidenced that lncRNA RP11-169D4.1 in LSCC tissues and cells was significantly downregulated. The overexpression of RP11-169D4.1 in LSCC cells suppresses the ability of cell migration and invasion and has the effect of antiproliferation and promoting cell apoptosis. It was confirmed that there was a binding site between miR-205-5p and RP11-169D4.1, and miR-205-5p could inhibit the expression of RP11-169D4.1. High expression of RP11-169D4.1 in SNU-899 (RRID: CVCL_5105) and SNU-46 (RRID: CVCL_5063) cells increased the level of E-cadherin but decreased the level of Snail2 and Vimentin. It is suggested that RP11-169D4.1 can inhibit the EMT process in LSCC cells (Zhao et al., 2017). The abnormal expression of Snail factor, especially Snail1 and Snail2, can be used as a common biomarker of EMT. Interestingly, Snail1, the transcriptional repressor of E-cadherin expression and a potent inducer of EMT, is able to induce the expression of *α*_v_ *β*_3_-integrin which is well known for its proinvasive functions and its localization in the invading front of cancers (Yilmaz and Christofori, 2009).

The expression of lncRNA ANRIL in LSCC increased, and it was negatively correlated with miR-181a. AMC-HN-8 and SNU-899 cell line was selected for *in vitro* experiment. The experimental results reported that ANRIL can directly combine with miR-181a sponge to counteract the inhibitory effect of miR-181a on Snail2 and play a positive role in regulating Snail2. Knockout of ANRIL gene or overexpression of miR-181a can greatly inhibit the proliferation, clonogenicity, invasion, migration, and EMT process of LSCC cells and elevate apoptosis (Hao et al., 2019).

LINC00467 is activated in HNSCC (head and neck squamous cell carcinoma) cells (HN6, SCC25, HN4, Cal27, SCC4), and silencing LINC00467 inhibited the growth, migration, and EMT process of HNSCC cells. In addition, LINC00467 overexpression competes to inhibit miR-299-5p and promotes USP48 (Ubiquitin specific protease-48) overexpression (Chen and Ding, 2020).

LINC00339 was proved to be an oncogene in LSCC cell line. LINC00339 silencing inhibited the proliferation, invasion, and EMT progression of LSCC cells. In addition, LINC00339 has a binding site to miR-145, and silencing LINC00339 elevates the transcription of miR-145 in LSCC cells (Liu and Duan, 2018).

LncRNA CCAT1 (colon cancer-associated transcript-1) was first discovered in colon cancer and facilitates the development of cancer. Compared with paracancerous tissues, lncRNA CCAT1 transcription was increased in LSCC tissues. Overexpression of CCAT1 encourages the proliferation, migration, invasion, colony formation, and cell cycle of HEp-2 cells. The ectopic expression of CCAT1 inhibits E-cadherin and enhances N-cadherin and Vimentin. It was also proved that the overexpression of CCAT1 inhibited let-7 and enhanced HMGA2 and Myc, the direct target genes of let-7 (Zhuang et al., 2016).

### LncRNA and Apoptosis

Apoptosis is particularly important for maintaining homeostasis of the internal environment. The genome that regulates apoptosis is large and complex, and it is also closely related to the generation and elimination of cancer. Suppressing the proliferation and promoting the apoptosis of cancer cells are the hot research directions at present.

It was identified that lncRNA MALAT1 (metastasis associated lung adenocarcinoma transcript 1) has been activated in tumor tissues of 24 patients with LSCC. Silencing MALAT1 in human laryngeal carcinoma cell line HEp-2 and pharynx cancer cell line FaDu (RRID: CVCL_1218) can induce apoptosis and inhibit cell proliferation, resulting in cell cycle arrest in the G1/G2 phase and significant relief in the S phase. Downregulation of MALAT1 expression can also inhibit the migration and invasion of HEp-2 and FaDu (Xu E. et al., 2019). Jiang et al. reported that high expression of MALAT1 can enhance the invasive ability of Tu 686 and LSC-1 (RRID: CVCL_9U29) cells, considerably improve the survival rate, facilitate proliferation, and inhibit apoptosis. Inhibition of MALAT1 expression in Tu 686 and LSC-1 cells could increase the expression of E-cadherin and alleviate the expression of N-cadherin/Vimentin (Jiang et al., 2019).

Compared with paracancerous tissues, lncRNA FTH1P3 (ferritin heavy chain-1 pseudogene-3) was substantially increased in LSCC tissues. In addition, the increased expression of FTH1P3 can promote the proliferation, migration, and invasion of LSCC cell line HEp-2 and Tu 212 and inhibit apoptosis. The overexpression of FTH1P3 leads to the increase of S phase cells and the decrease of G0/G1 phase cells (Yuan et al., 2019).

Abnormally high expressions of lncRNA MIR31HG and HIF1A were detected in LSCC tissues by microarray analysis. Additional experiments demonstrated that MIR31HG improved cell proliferation, upgraded cell cycle progression, and inhibited cell apoptosis *in vitro* and *in vivo*. Silencing MIR31HG promotes cell cycle arrest in G1 or S phase. Mechanism studies have shown that MIR31HG regulates LSCC cell cycle progression and apoptosis through cytoplasmic HIF1A and nuclear p21 (Wang et al., 2018).

It is known that lncRNA NEAT1 (nuclear paraspeckle assembly transcript 1) plays a key role in the occurrence and development of several tumors. It was confirmed that the level of NEAT1 in LSCC tissues was drastically higher than that in paracancerous tissues. In addition, silencing NEAT1 gene inhibited the proliferation of HEp-2 cells and induced apoptosis and cell cycle arrest in G1 phase. It was further revealed that silencing NEAT1 gene could inhibit the growth of LSCC transplanted tumor. Mechanism studies have demonstrated that NEAT1 upregulated CDK6 (cyclin-dependent kinase 6) through inhibiting the expression of miR-107 (Wang et al., 2016).

Previous research has proven that the transcription level of lncRNA NEF in tumor tissues of LSCC patients was lower than that of paracancerous normal tissue, and the level of serum NEF was considerably lower than that of healthy controls. Overexpression of NEF can inhibit the proliferation of human LSCC cell line [UM-SCC-17A (RRID: CVCL_7724)], improve apoptosis, and downregulate *β*-catenin. Wnt agonist has no significant effect on the expression of NEF, but Wnt agonist could reverse the effect of overexpression of NEF on proliferation and apoptosis of cancer cells (Cui X. et al., 2019).

LncRNA PVT1 was overexpressed in LSCC tissues and several kinds of human LSCC cell lines (Tu 686, Tu 177, and LSC-1). Silencing PVT1 significantly inhibited the proliferation of LSCC cells, promoted apoptosis, and reduced migration. *In vitro* experiments have shown that PVT1 facilitates the proliferation and migration of laryngeal squamous cell carcinoma by inhibiting the expression of miR-519d-3p (Zheng et al., 2019).

LncRNA SOX2-OT (Sox2 overlapping transcript) has been identified as an oncogene in various cancers. It must be pointed out that the overexpression of SOX2-OT in LSCC tissues was negatively correlated with the expression of PTEN. In addition, the overexpression of SOX2-OT promoted the proliferation, migration, invasion, and inhibition of apoptosis of LSCC cell lines [HEp-2 and UM-SCC-10A (RRID: CVCL_7713)] *in vitro*. Mechanism studies have shown that SOX2-OT interacts with EZH2 (enhancer of zeste homolog 2), recruits EZH2 to induce H3K27me3, and epigenetically inhibits PTEN expression in LSCC cells, thus promoting the development of LSCC (Tai et al., 2019).

### LSCC and Small Nucleolar RNA Host Genes

The small nucleolar RNA host genes (SNHGs) are a group of long noncoding RNAs, which are reported in many studies as being overexpressed in various cancers. With few exceptions, the SNHGs are recognized as inducing increased proliferation, cell cycle progression, invasion, and metastasis of cancer cells, which makes this class of transcripts a viable biomarker for cancer development and aggressiveness (Zimta et al., 2020).

It was demonstrated that lncRNA SNHG1 (small nucleolar RNA host gene 1) was substantially upregulated in LSCC tissues. SNHG1 gene knockout can inhibit the proliferation, migration, and invasion of AMC-HN-8 and HEp-2 and induce apoptosis. In addition, SNHG1 gene knockout can inhibit the growth and migration of LSCC xenografts in mice. Mechanism studies have shown that SNHG1 is the ceRNA of miR-375 and can improve the activity of YAP1. Moreover, YAP1 interacts with SNHG1 promoter to enhance the transcription of SNHG1 (Gao et al., 2018).

Previous studies have demonstrated that the transcription of SNHG1 in cancer tissues was greatly higher than that in paracancerous tissues. Silencing SNHG1 gene inhibits the proliferation of HEp-2 cells and promotes apoptosis. Additional evidence showed that SNHG1 gene knockout inhibits the invasion and migration of HEp-2 cells by inhibiting the EMT process and the expression of matrix metalloproteinase-2 (MMP-2) and MMP-9 (Lin et al., 2018).

Suppressing SNHG12 using siRNA inhibited proliferation and invasion and promoted apoptosis in the AMC-HN-8 LSCC cell line. Several recent researches have suggested and confirmed that SNHG12 acts on miR-129-5p and triggers its expression. Inhibition of miR-129-5p considerably increased proliferation and invasion of AMC-HN-8 cells and ameliorated the suppressive effects of si-SNHG12. miR129-5p was able to combine with the 3′UTR region of WW Domain-Containing E3 Ubiquitin Protein Ligase 1(WWP1), which is generally regarded as an E3 ubiquitin protein ligase. WWP1 was positively governed by SNHG12 and negatively regulated by miR-129-5p at the mRNA level and protein level. Overexpression of WWP1 drastically increased proliferation and invasion of laryngeal cancer cells. Moreover, when SNHG12 was suppressed, rescue of WWP1 restored the proliferation and invasion abilities of AMC-HN-8 cells (Li J. et al., 2019).

SNHG20 was found to be substantially suppressed in LSCC tissues or cells. The proliferation ability of AMC-HN-8 and HEp-2 cells with SNHG20 gene knockout was dramatically lessened. However, a recent study identified that there were binding sites between miR-140 and SNHG20, and there was a negative correlation between them. miR-140 is the target gene of SNHG20 (Li R. et al., 2019).

It was found that the expression of LncRNA Gas5 (growth arrest-specific 5) decreased in the LSCC tissue and Human LSCC cell lines [SNU-899 and SNU-1076 (RRID: CVCL_5006)], while miR-21 was on the contrary. In addition, ectopic expression of Gas5 significantly inhibited cell proliferation and facilitated cell apoptosis. Overexpression of miR-21 eliminated the inhibition of apoptosis and proliferation mediated by Gas5. Upregulated Gas5 can negatively modulate miR-21 and further regulate the target genes BAX and CDK6 of miR-21 (Lyu et al., 2019).

### LSCC and Homeobox (HOX) Transcript Antisense RNA

HOTAIR [Homeobox (HOX) transcript antisense RNA)] is an early discovery that transregulates the expression of HOX. LncRNA is about 2.2 kb, located between HOXC12 and HOXC11 genes. HOTAIR is upregulated in a variety of tumors and is closely related to poor prognosis. It plays an important role in tumor proliferation, invasion, and migration.

The expression level of HOTAIR in LSCC was considerably higher than that in the corresponding paracancerous nontumor tissues. siRNA-mediated HOTAIR gene knockout can reduce the invasiveness of HEp-2 cells *in vitro*, increase apoptosis, and significantly inhibit the growth of LSCC xenografts in mice. Indeed, several recent experiments have beautifully shown that PTEN methylation was dramatically increased in HEp-2 cells overexpressing HOTAIR, while PTEN methylation was markedly lessened in HEp-2 cells depleted HOTAIR. It is suggested that the carcinogenic effect of HOTAIR in LSCC is related to the promotion of PTEN methylation (Li et al., 2013).

In patients with early LSCC, the content of lncRNA PTCSC3 in plasma decreased. *In vitro*, the overexpression of PTCSC3 led to the inhibition of LSCC cell proliferation. In addition, in UM-SCC-17A cells, PTCSC3 inhibited the expression of STAT3 and HOTAIR, while STAT3 promoted the expression of HOTAIR. It is suggested that the inhibitory effect of PTCSC3 on the proliferation of cancer cells may be related to HOTAIR (Xiao et al., 2019a).

### LSCC and X Inactive-Specific Transcript

LncRNA XIST (X-inactive specific transcript) is dysregulated in the occurrence and development of a variety of cancers. Previous research has demonstrated that XIST gene is generally overexpressed in LSCC tissues. It is also worth noting that lncRNA XIST plays a key role in X chromosome inactivation (XCI). XCI is essential for proper development and cellular differentiation (Salama et al., 2019).

lncRNA XIST gene knockout can inhibit the proliferation, migration, and invasion of LSCC cell line HEp-2 and HEK293T (RRID: CVCL_0063). *In vivo*, XIST gene knockout can also inhibit the growth of LSCC xenografts in mice. XIST is the ceRNA of miR-124. In addition, EZH2 is the target gene of miR-124. It is confirmed that XIST modulate s the expression of EZH2 through competitive inhibition of miR-124 (Xiao et al., 2019b).

After knockout of XIST gene, the proliferation, colony formation, migration, and invasion of Tu 212 cells were dramatically inhibited, while apoptosis was significantly increased. Moreover, recent experimental evidence confirmed that XIST gene binds to the endogenous sponge of miR-144 and inhibits the expression of miR-144. IRS1 (insulin receptor substrate 1) is the target gene of miR-144, so XIST positively regulates the expression of IRS1. XIST gene knockout can also inhibit the growth of LSCC xenografts in mice (Cui et al., 2020).

### Other Types of lncRNA

It was confirmed that significant intensification of DLX6-AS1 in LSCC tissues and miR-376c had complementary binding sites with DLX6-AS1. Silencing DLX6-AS1 significantly inhibited the growth of HEp-2 cells and enhanced the transcription of miR-376c in HEp-2 cells at an unprecedented rate. Ectopic expression of miR-376c suppressed cell proliferation, cycle, and invasion of LSCC cell. Therefore, DLX6-AS1 triggers the development of LSCC by regulating the target gene miR-376c (Yang et al., 2019).

The role of LncRNA Loc285194 in several cancers is a tumor suppressor, and it confirmed the significant downregulation of loc285194 in patients with LSCC. There was a negative correlation between the transcription of Loc285194 and hexokinase 2 (HK-2) mRNA in LSCC patients but no correlation in healthy controls. In addition, it is confirmed that HK-2 mRNA is the downstream target of loc285194 in Human LSCC cell line UM-SCC-17A. *In vivo* experiments, the transcription of loc285194 was negatively correlated with tumor size but not with metastasis. Therefore, overexpression of loc285194 can inhibit HK-2 mRNA and the proliferation of LSCC cells (Gao Y. et al., 2019).

LncRNA HOXA11-AS was significantly upregulated in LSCC tissues. In addition, downregulation of HOXA11-AS can dramatically inhibit the growth, migration, and invasion of LSCC cell line AMC-HN-8 and HEp-2 (Qu et al., 2018). Knockdown of LINC00668 inhibits the proliferation, migration, and invasion of LSCC cell lines (Tu 177, Tu 21 2, Tu 686, and AMC-HN-8) (Zhao L. et al., 2019). Preliminary RNA-seq data suggested the upregulated expression of lncRNA MIR100HG and its inverse correlation with miR-204-5p, which was confirmed by the detection of cancer tissues in LSCC patients. Overexpression of MIR100HG promotes the proliferation, migration, and invasion of UM-SCC-17A cells. Further experiments identified that MIR100HG played its role by regulating the expression of miR-204-5p (Huang et al., 2019).

It was confirmed that the transcriptional level of lncRNA LOC554202 in LSCC tissues increased, while that of miR-31 alleviated. Ectopic expression of LOC554202 triggers the proliferation, cell cycle, and invasion of LSCC cells. Mechanism studies have shown that the high expression of LOC554202 plays the role of oncogenes by inhibiting miR-31, to facilitate the target gene RhoA of miR-31 (Yang et al., 2018).

Increased expression of lncRNA ST7-AS1 (suppressor of tumorigenicity 7 antisense RNA 1) was detected in both LSCC tissues and cell lines. Upregulation of ST7-AS1 expression enhanced the migration ability of Tu 212 and HEp-2 cells and can hasten the growth of LSCC xenografts in mice. ST7-AS1 could interact with CARM1, a well characterized protein arginine methyltransferase, and protect CARM1 from ubiquitin-dependent degradation. CARM1 can methylate Sox-2, a pluripotent transcription factor. Thus, ST7-AS1 might mediate its oncogenic effect by signaling through CARM1-Sox-2 axis to enhance Sox-2 self-association and transactivation activity (Qin et al., 2019). Overexpression of lncRNA TUG1 (taurine-upregulated gene 1) can promote the proliferation, invasion, and migration of HEp-2 cells. It was also found that silencing TUG1 significantly increased the expression of p53 mRNA and protein (Zhang J. et al., 2018).

lncRNA ZEB2-AS1 (zinc finger E-box-binding homeobox 2 antisense RNA 1) was abnormally highly expressed in both LSCC and LSCC cell lines (Tu 212, HEp-2 tissues). In addition, the overexpression of ZEB2-AS1 promotes the proliferation, migration, and invasion of LSCC cells. It is found that ZEB2-AS1 is the ceRNA of miR-6840-3p. MiR-6840-3p negatively controls PLXNB1. Therefore, overexpression of ZEB2-AS1 modulated the enhancement of PLXNB1 by blocking the expression of miR-6840-3p, which in turn promotes the progression of LSCC (Xu Q. et al., 2019).

It is reported that the transcription of lncRNA RGMB-AS1has been enhanced in many malignant tumors. The detection of increased expression of RGMB-AS1 in LSCC tissues also confirmed this. Silencing RGMB-AS1 inhibits the proliferation and invasion of HEp-2 and AMC-HN-8 cells *in vitro* and inhibits the growth of LSCC xenografts in mice. Mechanism research shows that lncRNA RGMB-AS1 is the ceRNA of miR-22. MiR-22 negatively regulates NLRP3. Overexpression of RGMB-AS1 competes to inhibit miR-22 and upregulates NLRP3 to facilitate the development of cancer (Xu and Xi, 2019).

miR143HG is decreased in LSCC and negatively correlated with miR-21. In Human LSCC cell line UM-SCC-17A, the overexpression of miR143HG led to the downregulation of miR-21 expression, while the overexpression of miR-21 did not affect miR143HG. Furthermore, recent studies also have confirmed that overexpression of miR143HG leads to an increase in miR-21 methylation and a decrease in the rate of migration and invasion of LSCC cells (Xun et al., 2019).

## Effects of Differentially Expressed lncRNA in LSCC on Signal Transduction Pathway

**Figure 3:**
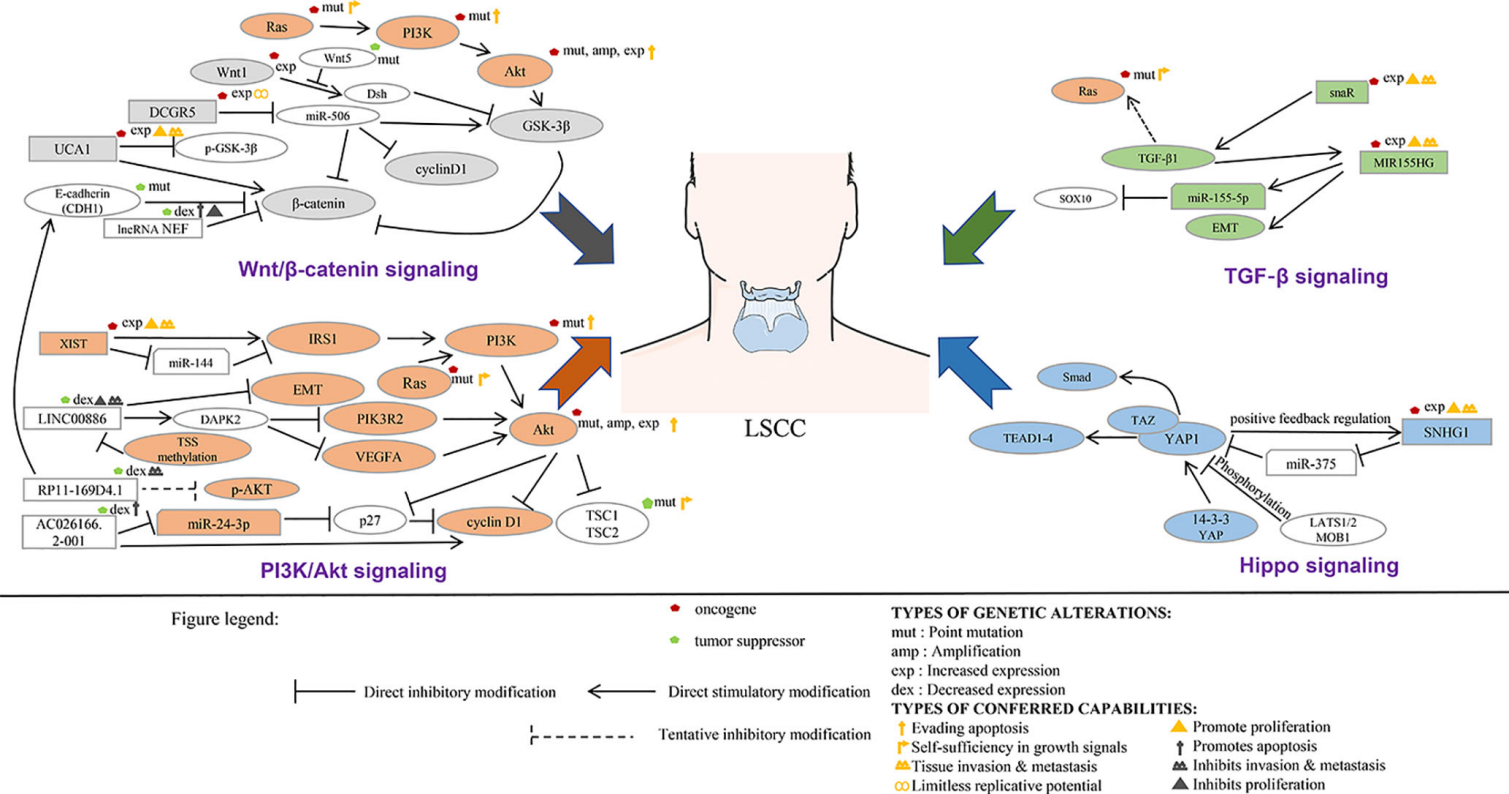
LncRNA involved in the signal transduction network of laryngeal squamous cell carcinoma.

Most proto-oncogenes, tumor suppressor genes, or their encoded proteins are components of complex cellular signal transduction networks and play an important role in signal transduction pathways. ncRNA can affect the occurrence and development of tumor by regulating the signaling pathways involved in its target genes. In tumor diseases, the research on lncRNA is mainly focused on the establishment of competing endogenous RNAs (ceRNAs) network and the regulation of miRNA expression. At present, the functional research of noncoding RNA mainly focused on microRNA and siRNA. The main reason is that the functional modes of these two kinds of small RNAs are through base pairing with the target genes, so it is relatively easy to find the targets of these small RNAs. For long noncoding RNAs, they usually form a complex secondary or even tertiary structure, so it is difficult to predict their targets sites. With the deepening of the study of lncRNA, the research on the function and structure of lncRNA tends to be combined with the signaling pathways involved in its regulation (**Figure 3**, **Table 3**). This part will analyze the regulatory relationship and role of lncRNA and tumor-related signaling pathways in the pathological process of LSCC.

**Table 3:** LncRNA involved in the signal transduction network of laryngeal squamous cell carcinoma.

lncRNA	Cells	Tissues	Expression in LSCCs	Related gene	Functional role	References (PMID)
lncRNA UCA1	AMC-HN-8	LSCC	Upregulated	Wnt/*β*-catenin signaling	Activation of Wnt/*β*-catenin signaling pathway promotes proliferation, invasion, and migration of LSCC cells	30679991
lncRNA NEF	UM-SCC-17A	LSCC	Downregulated	Wnt/*β*-catenin signaling	Inhibition of Wnt/*β*-catenin signaling pathway inhibits the proliferation of LSCC and promotes apoptosis	31186702
lncRNA DGCR5	HEp-2		Upregulated	miR-506/Wnt/*β*-catenin signaling	Promote CSC-like phenotype	30980388
lncRNA XIST	HEp-2, HEK293T	LSCC	Upregulated	miR-144/IRS1/PI3K/AKT signaling	Promote the ability of proliferation, colony formation, migration, and invasion	31894287
LINC00886	Tu 686, AMC-HN-8, Tu 177, 293 T	LSCC	Downregulated	VEGFA/PI3K/AKT signaling pathway/EMT	Overexpression significantly reduced cell proliferation, migration and invasion *in vitro*, and inhibited tumor growth *in vivo*	32111441
lncRNA RP11-169D4.1	SNU-899, SNU-46	LSCC	Downregulated	miR-205-5p/RP11-169D4.1/Cdh1/AKT signaling pathway	Reduced the ability of cell migration and invasion *in vitro*	28534968
lncRNA AC026166.2-001	AMC-HN-8, TU-212		Downregulated	miR-24-3p/p27/PI3K/AKT signaling	It can inhibit the proliferation and promote apoptosis of LSCC cells *in vitro* and *in vivo*	29463827/25243407
lncRNA SNHG1	AMC-HN-8, HEp-2	LSCC	Upregulated	miR-375/YAP1/HIPPO signaling pathway	Promote growth and transfer *in vivo*	30323965
lncRNA MIR155HG	Tu 686, Tu 177, AMC-HN-8, 293T	LSCC	Upregulated	miR-155-5p/TGF-*β*1/Sox10/TGF‐*β* signaling	Promote the progress of LSCC and EMT	31081043
lncRNA snaR	UM-SCC-17A	LSCC	Upregulated	TGF-*β*1/TGF-*β* signaling	Promote the proliferation, migration, and invasion of LSCC cells	30506952

### LncRNA in LSCC and Wnt/*β*-Catenin Signaling

The Wnt signaling pathway, which exists widely in invertebrates and vertebrates, is a kind of signaling pathway that is highly conserved in the process of species evolution. Wnt signaling plays an important role in early embryonic development, organogenesis, tissue regeneration, and other physiological processes. The stable conduction of the Wnt/*β*-catenin signaling pathway depends on the stability of *β*-catenin. The interaction between the *β*-catenin and LEF/TCF involves transcriptional regulators and histone modifiers, which in turn mediates excessive development and balance *in vivo* (Zhang H. et al., 2018). It is becoming more and more clear that the signaling pathway does not run in isolation but is closely intertwined. The Wnt/*β*-catenin signaling pathway works independently or in conjunction with other signaling pathways to induce appropriate cellular responses (Zhang H. et al., 2018).

The expression level of lncRNA UCA1 in tumor tissues of most LSCC patients was significantly higher than that in paracancerous normal tissue. Compared with the healthy control group, the level of serum UCA1 in patients with LSCC was considerably higher. UCA1 overexpression can promote the proliferation, migration, and invasion of LSCC cells. After overexpression of UCA1, there was no significant change in the expression level of GSK-3 *β* in AMC-HN-8 cells, but the phosphorylation level of GSK-3 *β* (p-GSK-3 *β*) lessened substantially, and the expression level of *β*-catenin increased at an unprecedented rate. However, UCA1 gene knockout and Wnt inhibitor therapy weakened the effect of UCA1 overexpression on the proliferation, migration, and invasion of LSCC. It is suggested that UCA1 promote the proliferation, migration and invasion of LSCC cells by activating Wnt/β-catenin signaling pathway (Sun et al., 2019).

LncRNA NEF inhibits proliferation and promotes apoptosis of LSCC cells by inhibiting Wnt/β-catenin signaling. Overexpression of NEF inhibited the proliferation of laryngeal squamous cell carcinoma cells, promoted apoptosis, and downregulated the expression of *β*-catenin. No significant effect of Wnt agonist on the expression of NEF was found, but Wnt agonist could reduce the effect of overexpression of NEF on proliferation and apoptosis of cancer cells (Cui X. et al., 2019).

Tumor stem cell (CSCs) has been considered to be an important cause of tumor recurrence. In HEp-2R (HEp-2R cells were harvested using repeated radiation) cells, lncRNA DGCR5 (DiGeorge syndrome critical region gene 5) expression is enhanced, while miR-506 is the opposite. In addition, silence of DGCR5 could inhibit the stemness and enhance the radiosensitivity of HEp-2R cells. Meanwhile, overexpression of miR-506 also suppressed the CSC-like traits, and the radiosensitivity was increased dramatically. The mechanism study shows that the target gene of DCGR5 is miR-506. DGCR5 inhibition could repress Wnt signaling activity by sponging miR-506. *In vivo* assays were performed and confirmed that DCGR5 depressed stemness of human laryngeal cancer cells through modulating miR-506 and Wnt signaling pathway (Tang and Shan, 2019).

### LncRNA in LSCC and PI3K/Akt Signaling

PI3K/Akt signaling plays a key role in regulating cell proliferation, growth, metabolism, and angiogenesis, and this signaling also interacts with other pathways, such as Ras/MAPK/NF-signaling B/Wnt pathway. Excessive activation of the PI3K/Akt/mTOR pathway leads to significant dysfunction of normal cells, which in turn leads to competitive growth and metabolism and angiogenesis. In this signaling pathway, PI3K activates phosphorylation and leads to the activation of Akt. Akt indirectly regulates cell proliferation, protein translation, and autophagy through mTOR activation. MTOR exists in the form of two multiprotein complexes of mTORC1 and mTORC2 and acts as a downstream effector of PI3K/Akt signaling pathway. The PI3K/Akt/mTOR pathway not only participates in cell proliferation and migration but also modulates intracellular protein synthesis to control metabolism. Abnormal activation of the PI3K/Ak/mTOR pathway usually occurs in malignant tumors such as gastric cancer, pancreatic cancer, and LSCC.

Lan et al. used Tu 177cells overexpressing LINC00886 to study the possible downstream target genes and signaling pathways. High-throughput sequencing technique was used to detect differential mRNA. GO and KEGG functional enrichment analyses are used to predict possible biological processes and signaling pathways mediated by the target mRNA. Six mRNAs were randomly selected to compensate the deficiencies of high-throughput sequencing using the qRT-PCR method. The relative expression of six mRNAs (VEGFA, PIK3R2, UNC5B, DAPK2, COL4A1, and EGR1) in Tu 177 cells overexpressing LINC00886 was consistent with the high-throughput sequencing results. Significant change in expression of VEGFA, PIK3R2, and DAPK2 overexpression of LINC00886 indicated that LINC00886 regulated biological function probably by participating in the VEGFA/PI3K/AKT signaling pathway (Lan et al., 2020).

Tumor suppressor gene AC026166.2-001inhibits the ability of proliferation and clone formation of LSCC cells, reduces migration, inhibits the G1-S cell cycle progression, and induces apoptosis. Mechanism studies show that AC026166.2-001 acts as a sponge of miR-24-3p and regulates the expression of p27 and cyclin D1 (Shen et al., 2018).

It was confirmed that there was a binding site between miR-205-5p and RP11-169D4.1, and RP11-169D4.1 could regulate the expression of E-cadherin. Ectopic transfection of RP11-169D4.1 resulted in a significant alleviation of the downstream signal molecule AKT in LSCC cells. Therefore, MiR-205-5p/RP11-169D4.1/E-cadherin/AKT signaling pathway is an important part of the molecular mechanism of EMT in laryngeal carcinoma (Zhao et al., 2017).

After knockout of XIST gene, the proliferation, colony formation, migration, and invasion of human LSCC Tu 212 cells were substantially inhibited, while apoptosis was considerably increased. XIST gene knockout can also inhibit the growth of LSCC xenografts in mice. Further studies showed that XIST gene binds to the endogenous sponge of miR-144 and inhibits the expression of miR-144. IRS1 is the target gene of miR-144, so XIST positively regulates IRS1. IRS1 was reported to be involved in regulating the PI3K/AKT signaling pathways. XIST gene knockout drastically inhibited the activation of PI3K/AKT pathway in Tu 212 cells (Cui et al., 2020).

HOTAIR is abnormally high expressed in LSCC, which promotes the occurrence and development of cancer. Further experiments identified that PTEN methylation was dramatically increased in HEp-2 cells overexpressing HOTAIR, while PTEN methylation was substantially declined in HEp-2 cells depleted HOTAIR. Studies have shown that PTEN can inhibit the PI3K signaling pathway (Li et al., 2013). Previous research has demonstrated that PTEN exerts its role as a tumor suppressor by negatively regulating the PI3K/PKB/Akt signaling pathway (Stambolic et al., 1998). Other studies have shown that SOX2-OT interacts with EZH2, recruits EZH2 to induce H3K27me3, and suppresses the expression of PTEN in LSCC cells in epigenetics, thus promoting the development of LSCC (Tai et al., 2019).

### LncRNA in LSCC and TGF-*β* Signaling

The TGF-*β* signaling pathway plays a key role in the growth, differentiation, and apoptosis of cells and tissues. The effect of TGF-*β* signaling on tumor cells can be found in almost all tumor cells. In addition, TGF-*β* promotes the expression of extracellular matrix (ECM) such as collagen and fibronectin and inhibits the degradation of ECM, which plays an important role in cell morphogenesis, proliferation, and differentiation.

LncRNA snaR is a characterized oncogenic lncRNA in triple negative breast cancer and ovarian cancer. There was a positive correlation between plasma snaR level and TGF-*β*1 in patients with LSCC, but not in healthy controls. *In vitro* experiments confirmed that overexpression of snaR induced the increase of TGF-*β*1 and enhanced the proliferation, migration, and invasion of LSCC cell line UM-SCC-17A (Liang et al., 2018).

MIR155HG and miR-155-5p were remarkably upregulated in LSCC tissues, and the synergistic effect of miR-155-5P and MIR155HG elevated the proliferation, migration, and invasion of LSCC cells. It has been discovered that the overexpression of miR-155-5p reduces the transcription and translation of SOX10, and SOX10 is the target gene of miR-155-5p. To further determine whether MIR155HG and miR-155-5p were involved in TGF-*β*-induced EMT, their expression was detected by RT-qPCR analysis. The results indicated that the levels of MIR155HG and miR-155-5p were upregulated after the TU177 cells were treated with TGF-β. *In vitro* experiments confirmed that MIR155HG and miR155-5p were upregulated under the induction of TGF-*β* and synergistically promoted the process of EMT (Cui W. et al., 2019).

### LncRNA in LSCC and Hippo Signaling

Hippo signaling is an evolutionarily conserved pathway that controls organ size by regulating cell proliferation, apoptosis, and stem cell self-renewal. In addition, disorders in the Hippo pathway can lead to the development of cancer. The core of the Hippo pathway is a kinase cascade in which Mst1/2 (a homolog of Drosophila Hippo) kinase and SAV1 form a complex that phosphorylates and activates LATS1/2. LATS1/2 kinase in turn phosphorylates and inhibits transcriptional coactivators YAP and TAZ. After dephosphorylation, YAP/TAZ is transported to the nucleus and interacts with TEAD1–4 and other transcription factors to induce gene expression that drives cell proliferation and inhibits apoptosis.

LncRNA SNHG1 was significantly upregulated in LSCC tissues. SNHG1 gene knockout can inhibit the proliferation, migration, and invasion of AMC-HN-8 and HEp-2 and induce apoptosis. In addition, SNHG1 gene knockout can inhibit the growth and migration of LSCC xenografts in mice. Mechanism studies have shown that SNHG1 is the ceRNA of miR-375 and exalts YAP1 and the activity of hippopotamus signal. Further research reveals that YAP1 interacts with the SNHG1 promoter to enhance the transcription of the latter, suggesting that there is a positive feedback regulation between YAP1 and SNHG1 (Gao et al., 2018).

### LncRNA in LSCC and Other Signal Transduction Pathways

Gao et al. used to explore the transcriptional regulation of miR-145-5p in LSCC. The miR-145-5p mimic was transfected into the LSCC cell line Tu 177 to obtain LSCC cells overexpressing miR-145-5p. Microarray analysis showed that 26 miRNAs, 248 mRNAs, 1,118 lncRNAs and 382 circRNAs were differentially expressed in miR-145-5p overexpressed LSCC cells. A total of 675 differentially expressed miRNA target genes were identified. These miRNAs involve ontology (GO) terminology of cell adhesion-related genes and MAPK and FoxO signaling pathways. In addition, 149 ceRNAs related to apoptosis, Wnt signaling pathway, and metabolic pathway were predicted. To verify whether miR-145-5p affects these signaling pathways, they monitored the expression of some key genes involved in MAPK and FoxO signaling pathways by qPCR. Compared to that of negative control group, MAP2K1, MAP3K1 (MAPK signaling pathway), FOXO1, TGFB1, AGAP2, SMAD3, and NLK (FoxO signaling pathway) were downregulated drastically in miR-145-5p overexpressed LSCC cells, indicating that miR-145-5p affected these pathways (Gao W. et al., 2019).

## Differentially Expressed lncRNA in LSCC and Drug Therapy

Although great progress has been made in chemotherapy, radiotherapy, and molecular targeted therapy in modern medicine in recent years, the prognosis of patients is still not good. In order to improve the overall survival rate of patients with advanced LSCC, it is urgent to reveal the potential mechanism of chemotherapy resistance in laryngeal squamous cell carcinoma and find new biomarkers for targeted therapy.

### LncRNA in LSCC and Chemotherapy

MALAT1 expression was higher in the LSCC tissues than in the adjacent normal tissues. For another, the expression of MALAT1 was also unusually elevated within the Tu 686, Tu 177, AMC-HN-8, and LSC-1 cell lines relative to NHBEC cell line. The Tu 686 cell line therein excelled in resisting the growth-curbing effects of 5-fluorouracil (IC50 = 20.44 μmol/L), paclitaxel (IC50 = 35.86 μg/L), and vincristine (IC50 = 0.12 μmol/L) when compared with the Tu 177, AMC-HN-8, and LSC-1 cell line. Moreover, there seemed great potential for overexpressed MALAT1 to enhance the chemo-resistance of both the Tu 686 and LSC-1 cell lines. Not only that, silencing of MALAT1 tended to undermine the proliferative and metastatic power of Tu 686 and LSC-1 cell lines (Jiang et al., 2019).

Chen et al. evaluated the effect of cisplatin and paclitaxel on lncRNA specifically expressed in LSCC by chemotherapy experiment. 37 candidate lncRNAs related to cancer were selected. Identification and confirmation suggested that in tumor samples, the relative expression of lncRNA CDKN2B-AS1, HOTAIR, and MALAT1 was relatively high, while the transcription level of lncRNA RRP1B and SRA1 was relatively low, a total of five significant lncRNAs. The transcription level of lncRNA CDKN2B-AS1, HOTAIR, and MALAT1 declined clearly with the increase of cisplatin and paclitaxel concentration and treatment time. It is suggested that cisplatin and paclitaxel target the important lncRNA in LSCC (Chen et al., 2014).

Zheng et al. reported that inhibiting HOTAIR could stimulate EZH2, upgrade the proliferation of AMC-HN8 cells, and increase the sensitivity of LSCC cells to cis-platinum. At the same time, it was found that the transcription of HOTAIR in HEp-2 and AMC-HN-8 cells changed after being cultured with cis-platinum and paclitaxel and suggesting that the abnormal expression of HOTAIR has a certain significance in drug resistance of cancer cells (Zheng et al., 2017).

Li et al. identified that lncRNA FOXD2-AS1 regulates LSCC therapeutic resistance by augmenting LSCC stemness. LSCC chemotherapy-resistant patients showed increased FOXD2-AS1 expression compared with that in the chemotherapysensitive patients, which predicted poor prognosis. Gain- or loss-of-function experiments have disclosed that upregulated FOXD2-AS1 maintained cancer stemness, reducing the response to chemotherapy, while FOXD2-AS1 downregulation had the opposite effects. FOXD2-AS1 acted as a scaffold for STAT3 and PRMT5, promoting STAT3 transcriptional activity, which is essential to maintain cancer stemness and facilitate chemotherapeutic resistance. Interfering with FOXD2-AS1 using short hairpin RNA rescued LSCC’s chemotherapeutic sensitivity. Thus, FOXD2-AS1 promotes LSCC chemotherapeutic resistance and is an upstream activator of STAT3, making FOXD2-AS1 a potential therapeutic target to improve the chemotherapy effect in LSCC patients (Li et al., 2020).

### LncRNA in LSCC and Radiation Therapy

In addition, significant upregulation of DGCR5 was observed in HEp-2R cells. Silencing DGCR5 can inhibit the proliferation of HEp-2R cells and make them sensitive to radiation. miR-195 is lowly expressed in HEp-2R cells and can inhibit the proliferation of HEp-2 cells. The study of machine mechanism shows that miR-195 is the downstream target of DGCR5. In addition, the overexpression of miR-195 greatly increased the radiosensitivity of HEp-2R cells. Upregulation of DGCR5 can reverse the effect of miR-195. Finally, *in vivo* experiments show that DGCR5 can promote the radiation resistance of LSCC cells by combining with sponge miR-195 (Tang et al., 2019).

## Conclusion

Most lncRNAs play a particularly important role in the proliferation, migration, invasion, and apoptosis of tumor. It is the abnormal expression of these genes that often leads to the deterioration of tumor. Analyzing the regulatory network of ncRNA and clarifying the function of ncRNA may be a major breakthrough in uncovering the mystery of huge human noncoding genes. In the research on the biological function of lncRNA in LSCC, the research results mainly focus on the effect of lncRNA on tumor proliferation, EMT process, tumor apoptosis and so on. The existing studies have not clarified the fundamental cause of inducing the abnormal expression of many lncRNAs in LSCC. It is also worth noting that there are few researches on the influence of LSCC inducing factors on lncRNA. In LSCC, there are few studies on the effect of drugs on lncRNA. In the process of drug development, to assist in the study of the positive effects of drugs on lncRNA-specific expression of LSCC may shorten the process of drug development. The study of lncRNA should be increased in the research of drugs that facilitate spontaneous apoptosis and inhibit invasion.

At present, the main task is still to discover more ncRNA and its biological functions and also to explore the relationship between them and cellular signal transduction. The study is long and painstaking, far more complex than the Genome Project. At present, it is reliable to use abnormally expressed ncRNA in cancer as a marker of diagnosis and prognosis, which not only indicates the importance of ncRNA function research, but also indicates that the current research is still in its infancy. The complex secondary structure and tertiary structure of lncRNA are not clear, its function is no longer only achieved by simple base complementation. The current research is more like the blind touch, unable to understand the structure and function of ncRNA in detail, and the treatment developed for ncRNA is even more on paper. Therefore, it is urgent to develop a more effective research model for ncRNA.

Not only that, ncRNA is poorly conserved among different species, and there are individual differences among the same species. Individualized cancer therapy is also in the research. On this basis, integrating a large number of statistically significant patients and determining internal and external factors will not only contribute to the study of ncRNA, but also contribute to the development of new diagnosis and targeted therapy strategies based on ncRNA. It seems very promising to bring a new paradigm for cancer research and may become the main treatment strategy for cancer treatment in the near future.

## Author Contributions

WL was responsible for literature review and writing. YC was responsible for the correction. XN was responsible for proofreading, literature review, and correction.

## Funding

This work was supported by the National Natural Science Foundation of China (No. 81960741,81560712), the Joint Fund Project of the Science and Technology Office of Guizhou Province (No.LH-2014-7566), the Major project of Science and Technology Department of Guizhou Province (No.2015-6010), Young science foundation of Guizhou Provincial Department of Education (No.KY-2015-439), 2016 Zunyi 15851 talents elite project funding (No.2015019), 2011 Collaborative Innovation Center of Guizhou Traditional Chinese Medicine and Ethnic medicine (No. Qian jiao ke yan fa 2012-311), Guizhou Provincial Administration of Traditional Chinese Medicine funding (No.QZYY2017-080). Special Funding for Postdoctoral Research Projects in Chongqing (No.Xm2019061), Guizhou Provincial Natural Science Foundation (QKH-J-2020-1Z070).

## Conflict of Interest

The authors declare that the research was conducted in the absence of any commercial or financial relationships that could be construed as a potential conflict of interest.

## References

[B1] Bhan A. Soleimani M. Mandal S. S. (2017). Long Noncoding RNA and Cancer: A New Paradigm. Cancer Res. 77 (15), 3965–3981. 10.1158/0008-5472.Can-16-2634 28701486 PMC8330958

[B2] Chen Y. Ding Y. (2020). LINC00467 enhances head and neck squamous cell carcinoma progression and epithelial-mesenchymal transition process via miR-299-5p/ubiquitin specific protease-48 axis. J. Gene Med. 22 (7), e3184. 10.1002/jgm.3184 32159247

[B3] Chen H. Xin Y. Zhou L. Huang J. M. Tao L. Cheng L. (2014). Cisplatin and paclitaxel target significant long noncoding RNAs in laryngeal squamous cell carcinoma. Med. Oncol. 31 (11):246. 10.1007/s12032-014-0246-7 25257554

[B4] Cui W. Meng W. Zhao L. Cao H. Chi W. Wang B. (2019). TGF-beta-induced long non-coding RNA MIR155HG promotes the progression and EMT of laryngeal squamous cell carcinoma by regulating the miR-155-5p/SOX10 axis. Int. J. Oncol. 54 (6), 2005–2018. 10.3892/ijo.2019.4784 31081043 PMC6521927

[B5] Cui X. Fang N. Cui Y. Xiao D. Wang X. (2019). Long non-coding RNA NEF inhibits proliferation and promotes apoptosis of laryngeal squamous cell carcinoma cells by inhibiting Wnt/beta-catenin signaling. Oncol. Lett. 17 (6), 4928–4934. 10.3892/ol.2019.10150 31186702 PMC6507471

[B6] Cui C. L. Li Y. N. Cui X. Y. Wu X. (2020). lncRNA XIST promotes the progression of laryngeal squamous cell carcinoma by sponging miR144 to regulate IRS1 expression. Oncol. Rep. 43 (2), 525–535. 10.3892/or.2019.7438 31894287 PMC6967080

[B7] Gao L. Cao H. Cheng X. (2018). A positive feedback regulation between long noncoding RNA SNHG1 and YAP1 modulates growth and metastasis in laryngeal squamous cell carcinoma. Am. J. Cancer Res. 8 (9), 1712–1724. 30323965 PMC6176179

[B8] Gao W. Zhang Y. Niu M. Bo Y. Li H. Xue X. (2019). Identification of miR-145-5p-Centered Competing Endogenous RNA Network in Laryngeal Squamous Cell Carcinoma. Proteomics 19 (21-22), e1900020. 10.1002/pmic.201900020 31169343

[B9] Gao Y. Wang Z. Tong J. Zheng Y. (2019). LncRNA loc285194 inhibits tumor growth of laryngeal squamous cell carcinoma cells by downregulating hexokinase 2. Exp. Ther. Med. 18 (4), 2378–2384. 10.3892/etm.2019.7761 31555348 PMC6755266

[B10] Gupta R. A. Shah N. Wang K. C. Kim J. Horlings H. M. Wong D. J. (2010). Long non-coding RNA HOTAIR reprograms chromatin state to promote cancer metastasis. Nature 464 (7291), 1071–1076. 10.1038/nature08975 20393566 PMC3049919

[B11] Guttman M. Amit I. Garber M. French C. Lin M. F. Feldser D. (2009). Chromatin signature reveals over a thousand highly conserved large non-coding RNAs in mammals. Nature 458 (7235), 223–227. 10.1038/nature07672 19182780 PMC2754849

[B12] Hao Y. R. Zhang D. J. Fu Z. M. Guo Y. Y. Guan G. F. (2019). Long non-coding RNA ANRIL promotes proliferation, clonogenicity, invasion and migration of laryngeal squamous cell carcinoma by regulating miR-181a/Snai2 axis. Regener. Ther. 11, 282–289. 10.1016/j.reth.2019.07.007 PMC6813643 31667207

[B13] Huang Y. Zhang C. Zhou Y. (2019). LncRNA MIR100HG promotes cancer cell proliferation, migration and invasion in laryngeal squamous cell carcinoma through the downregulation of miR-204-5p. Onco Targets Ther. 12, 2967–2973. 10.2147/ott.S202528 31114240 PMC6489621

[B14] Jiang Q. Liu S. Hou L. Guan Y. Yang S. Luo Z. (2019). The implication of LncRNA MALAT1 in promoting chemo-resistance of laryngeal squamous cell carcinoma cells. J. Clin. Lab. Anal. 34 (4), e23116. 10.1002/jcla.23116 31837057 PMC7171298

[B15] Kogo R. Shimamura T. Mimori K. Kawahara K. Imoto S. Sudo T. (2011). Long noncoding RNA HOTAIR regulates polycomb-dependent chromatin modification and is associated with poor prognosis in colorectal cancers. Cancer Res. 71 (20), 6320–6326. 10.1158/0008-5472.Can-11-1021 21862635

[B16] Lan L. Cao H. Chi W. Meng W. Zhao L. Cui W. (2020). Aberrant DNA hypermethylation-silenced LINC00886 gene accelerates malignant progression of laryngeal carcinoma. Pathol. Res. Pract. 216 (4), 152877. 10.1016/j.prp.2020.152877 32111441

[B17] Li D. Feng J. Wu T. Wang Y. Sun Y. Ren J. (2013). Long intergenic noncoding RNA HOTAIR is overexpressed and regulates PTEN methylation in laryngeal squamous cell carcinoma. Am. J. Pathol. 182 (1), 64–70. 10.1016/j.ajpath.2012.08.042 23141928

[B18] Li J. Sun S. Chen W. Yuan K. (2019). Small Nucleolar RNA Host Gene 12 (SNHG12) Promotes Proliferation and Invasion of Laryngeal Cancer Cells via Sponging miR-129-5p and Potentiating WW Domain-Containing E3 Ubiquitin Protein Ligase 1 (WWP1) Expression. Med. Sci. Monit. 25, 5552–5560. 10.12659/msm.917088 31348766 PMC6681687

[B19] Li Y. Xu J. Guo Y. N. Yang B. B. (2019). LncRNA SNHG20 promotes the development of laryngeal squamous cell carcinoma by regulating miR-140. Eur. Rev. Med. Pharmacol. Sci. 23 (8), 3401–3409. 10.26355/eurrev_201904_17704 31081112

[B20] Li R. Chen S. Zhan J. Li X. Liu W. Sheng X. (2020). Long noncoding RNA FOXD2-AS1 enhances chemotherapeutic resistance of laryngeal squamous cell carcinoma via STAT3 activation. Cell Death Dis. 11 (1), 41. 10.1038/s41419-020-2232-7 31959918 PMC6971019

[B21] Liang K. Yang Y. Zha D. Yue B. Qiu J. Zhang C. (2018). Overexpression of lncRNA snaR is correlated with progression and predicts poor survival of laryngeal squamous cell carcinoma. J. Cell Biochem. 120 (5), 8492–8498. 10.1002/jcb.28136 30506952

[B22] Lin S. X. Jiang H. Xiang G. Z. Zhang W. R. Weng Y. H. Qiu F. D. (2018). Up-regulation of long non-coding RNA SNHG1 contributes to proliferation and metastasis in laryngeal squamous cell carcinoma. Eur. Rev. Med. Pharmacol. Sci. 22 (5), 1333–1341. 10.26355/eurrev_201803_14475 29565491

[B23] Liu S. Duan W. (2018). Long noncoding RNA LINC00339 promotes laryngeal squamous cell carcinoma cell proliferation and invasion via sponging miR-145. J. Cell Biochem. 120 (5), 8272–8279. 10.1002/jcb.28110 30485513

[B24] Lyu K. Xu Y. Yue H. Li Y. Zhao J. Chen L. (2019). Long Noncoding RNA GAS5 Acts As A Tumor Suppressor In Laryngeal Squamous Cell Carcinoma Via miR-21. Cancer Manag. Res. 11, 8487–8498. 10.2147/cmar.S213690 31572003 PMC6756574

[B25] Meng W. Cui W. Zhao L. Chi W. Cao H. Wang B. (2019). Aberrant methylation and downregulation of ZNF667-AS1 and ZNF667 promote the malignant progression of laryngeal squamous cell carcinoma. J. BioMed. Sci. 26 (1), 13. 10.1186/s12929-019-0506-0 30684967 PMC6347788

[B26] Pandey R. R. Mondal T. Mohammad F. Enroth S. Redrup L. Komorowski J. (2008). Kcnq1ot1 antisense noncoding RNA mediates lineage-specific transcriptional silencing through chromatin-level regulation. Mol. Cell 32 (2), 232–246. 10.1016/j.molcel.2008.08.022 18951091

[B27] Qin H. Xu J. Gong L. Jiang B. Zhao W. (2019). The long noncoding RNA ST7-AS1 promotes laryngeal squamous cell carcinoma by stabilizing CARM1. Biochem. Biophys. Res. Commun. 512 (1), 34–40. 10.1016/j.bbrc.2019.02.057 30853182

[B28] Qu L. Jin M. Yang L. Sun C. Wang P. Li Y. (2018). Expression of long non-coding RNA HOXA11-AS is correlated with progression of laryngeal squamous cell carcinoma. Am. J. Transl. Res. 10 (2), 573–580. 29511452 PMC5835823

[B29] Salama E. A. Adbeltawab R. E. El Tayabi H. M. (2019). XIST and TSIX: Novel cancer immune biomarkers in PD-L1-overexpressing breast cancer patients. Front. Oncol. 9, 1459. 10.3389/fonc.2019.01459 31998636 PMC6966712

[B30] Shen Z. Li Q. Deng H. Lu D. Song H. Guo J. (2014). Long non-coding RNA profiling in laryngeal squamous cell carcinoma and its clinical significance: potential biomarkers for LSCC. PloS One 9 (9), e108237. 10.1371/journal.pone.0108237 25243407 PMC4171522

[B31] Shen Z. Hao W. Zhou C. Deng H. Ye D. Li Q. (2018). Long non-coding RNA AC026166.2-001 inhibits cell proliferation and migration in laryngeal squamous cell carcinoma by regulating the miR-24-3p/p27 axis. Sci. Rep. 8 (1), 3375. 10.1038/s41598-018-21659-5 29463827 PMC5820272

[B32] Stambolic V. Suzuki A. de la Pompa J. L. Brothers G. M. Mirtos C. Sasaki T. (1998). Negative regulation of PKB/Akt-dependent cell survival by the tumor suppressor PTEN. Cell 95 (1), 29–39. 10.1016/s0092-8674(00)81780-8 9778245

[B33] Steuer C. E. El-Deiry M. Parks J. R. Higgins K. A. Saba N. F. (2017). An update on larynx cancer. CA Cancer J. Clin. 67 (1), 31–50. 10.3322/caac.21386 27898173

[B34] Sun S. Gong C. Yuan K. (2019). LncRNA UCA1 promotes cell proliferation, invasion and migration of laryngeal squamous cell carcinoma cells by activating Wnt/beta-catenin signaling pathway. Exp. Ther. Med. 17 (2), 1182–1189. 10.3892/etm.2018.7097 30679991 PMC6327537

[B35] Tai Y. Ji Y. Liu F. Zang Y. Xu D. Ma S. (2019). Long noncoding RNA SOX2-OT facilitates laryngeal squamous cell carcinoma development by epigenetically inhibiting PTEN via methyltransferase EZH2. IUBMB Life 71 (9), 1230–1239. 10.1002/iub.2026 30811870

[B36] Tang T. Shan G. (2019). DGCR5 promotes cancer stem cell-like properties of radioresistant laryngeal carcinoma cells by sponging miR-506 via Wnt pathway. J. Cell Physiol. 234 (10), 18423–18431. 10.1002/jcp.28478 30980388

[B37] Tang T. Shan G. Zeng F. (2019). Knockdown of DGCR5 enhances the radiosensitivity of human laryngeal carcinoma cells via inducing miR-195. J. Cell Physiol. 234 (8), 12918–12925. 10.1002/jcp.27958 30549038 PMC13483208

[B38] Wang P. Wu T. Zhou H. Jin Q. He G. Yu H. (2016). Long noncoding RNA NEAT1 promotes laryngeal squamous cell cancer through regulating miR-107/CDK6 pathway. J. Exp. Clin. Cancer Res. 35, 22. 10.1186/s13046-016-0297-z 26822763 PMC4731996

[B39] Wang R. Ma Z. Feng L. Yang Y. Tan C. Shi Q. (2018). LncRNA MIR31HG targets HIF1A and P21 to facilitate head and neck cancer cell proliferation and tumorigenesis by promoting cell-cycle progression. Mol. Cancer 17 (1), 162. 10.1186/s12943-018-0916-8 30458787 PMC6247607

[B40] Wang B. Zhao L. Chi W. Cao H. Cui W. Meng W. (2019). Aberrant methylation-mediated downregulation of lncRNA SSTR5-AS1 promotes progression and metastasis of laryngeal squamous cell carcinoma. Epigenet. Chromatin 12 (1), 35. 10.1186/s13072-019-0283-8 PMC6563380 31196171

[B41] Wang H. Qian J. Xia X. Ye B. (2020). Long non-coding RNA OIP5-AS1 serves as an oncogene in laryngeal squamous cell carcinoma by regulating miR-204-5p/ZEB1 axis. Naunyn Schmiedebergs Arch. Pharmacol. 10.1007/s00210-020-01811-7 32009213

[B42] Xiao D. Cui X. Wang X. (2019a). LncRNA PTCSC3 inhibits cell proliferation in laryngeal squamous cell carcinoma by down-regulating lncRNA HOTAIR. Biosci. Rep. 39 (6), BSR20182362. 10.1042/bsr20182362 31171714 PMC6597852

[B43] Xiao D. Cui X. Wang X. (2019b). Long noncoding RNA XIST increases the aggressiveness of laryngeal squamous cell carcinoma by regulating miR-124-3p/EZH2. Exp. Cell Res. 381 (2), 172–178. 10.1016/j.yexcr.2019.04.034 31071316

[B44] Xu Z. Xi K. (2019). LncRNA RGMB-AS1 promotes laryngeal squamous cell carcinoma cells progression via sponging miR-22/NLRP3 axis. BioMed. Pharmacother. 118, 109222. 10.1016/j.biopha.2019.109222 31351424

[B45] Xu E. Liang X. Ji Z. Zhao S. Li L. Lang J. (2019). Blocking long noncoding RNA MALAT1 restrained the development of laryngeal and hypopharyngeal carcinoma. Eur. Arch. Otorhinolaryngol. 277 (2), 611–621. 10.1007/s00405-019-05732-x 31792655 PMC6981317

[B46] Xu Q. Liu H. Yu B. Chen W. Zhai L. Li X. (2019). Long noncoding RNA ZEB2-AS1 facilitates laryngeal squamous cell carcinoma progression by miR-6840-3p/PLXNB1 axis. Onco Targets Ther. 12, 7337–7345. 10.2147/ott.S212749 31564916 PMC6735660

[B47] Xun W. Cen W. Dahai Y. Huaqing W. Jiping S. Mengzhu G. (2019). LncRNA miR143HG suppresses miR-21 through methylation to inhibit cell invasion and migration. Laryngoscope. 10.1002/lary.28474 31872875

[B48] Yang S. Wang J. Ge W. Jiang Y. (2018). Long non-coding RNA LOC554202 promotes laryngeal squamous cell carcinoma progression through regulating miR-31. J. Cell Biochem. 119 (8), 6953–6960. 10.1002/jcb.26902 29737563

[B49] Yang Q. Sun J. Ma Y. Zhao C. Song J. (2019). LncRNA DLX6-AS1 promotes laryngeal squamous cell carcinoma growth and invasion through regulating miR-376c. Am. J. Transl. Res. 11 (11), 7009–7017. 31814904 PMC6895517

[B50] Yilmaz M. Christofori G. (2009). EMT, the cytoskeleton, and cancer cell invasion. Cancer Metastasis Rev. 28, 15–33. 10.1007/s10555-008-9169-0 19169796

[B51] Yuan H. Jiang H. Wang Y. Dong Y. (2019). Increased expression of lncRNA FTH1P3 predicts a poor prognosis and promotes aggressive phenotypes of laryngeal squamous cell carcinoma. Biosci. Rep. 39 (6), BSR20181644. 10.1042/bsr20181644 31142627 PMC6580104

[B52] Zhang H. Nie X. Shi X. Zhao J. Chen Y. Yao Q. (2018). Regulatory Mechanisms of the Wnt/beta-Catenin Pathway in Diabetic Cutaneous Ulcers. Front. Pharmacol. 9, 1114. 10.3389/fphar.2018.01114 30386236 PMC6199358

[B53] Zhang Z. Wang X. Cao S. Han X. Wang Z. Zhao X. (2018). The Long Noncoding RNA TUG1 Promotes Laryngeal Cancer Proliferation and Migration. Cell Physiol. Biochem. 49 (6), 2511–2520. 10.1159/000493876 30261503

[B54] Zhao J. Lv K. Li Z. H. Wu J. Gao W. Wong T. S. (2017). Functional significance of the long non-coding RNA RP11-169D4.1 as a metastasis suppressor in laryngeal squamous cell carcinoma by regulating CDH1. Oncol. Rep. 38 (1), 211–220. 10.3892/or.2017.5645 28534968

[B55] Zhao L. Cao H. Chi W. Meng W. Cui W. Guo W. (2019). Expression profile analysis identifies the long non-coding RNA landscape and the potential carcinogenic functions of LINC00668 in laryngeal squamous cell carcinoma. Gene 687, 47–55. 10.1016/j.gene.2018.11.020 30415008

[B56] Zhao Y. Q. Liu X. B. Xu H. Liu S. Wang J. M. (2019). MEG3 inhibits cell proliferation, invasion and epithelial-mesenchymal transition in laryngeal squamous cell carcinoma. Eur. Rev. Med. Pharmacol. Sci. 23 (5), 2062–2068. 10.26355/eurrev_201903_17247 30915750

[B57] Zheng J. Xiao X. Wu C. Huang J. Zhang Y. Xie M. (2017). The role of long non-coding RNA HOTAIR in the progression and development of laryngeal squamous cell carcinoma interacting with EZH2. Acta Otolaryngol. 137 (1), 90–98. 10.1080/00016489.2016.1214982 27542077

[B58] Zheng X. Zhao K. Liu T. Liu L. Zhou C. Xu M. (2019). Long noncoding RNA PVT1 promotes laryngeal squamous cell carcinoma development by acting as a molecular sponge to regulate miR-519d-3p. J. Cell Biochem. 120 (3), 3911–3921. 10.1002/jcb.27673 30304557

[B59] Zhuang K. Wu Q. Jiang S. Yuan H. Huang S. Li H. (2016). CCAT1 promotes laryngeal squamous cell carcinoma cell proliferation and invasion. Am. J. Transl. Res. 8 (10), 4338–4345. 27830017 PMC5095326

[B60] Zimta A. A. Tigu A. B. Braicu C. Stefan C. Ionescu C. Berindan-Neagoe I. (2020). An Emerging Class of Long Non-coding RNA With Oncogenic Role Arises From the snoRNA Host Genes. Front. Oncol. 10, 389. 10.3389/fonc.2020.00389 32318335 PMC7154078

